# Sequential Cobalt Magnetization Collapse in ErCo_2_: Beyond the Limits of Itinerant Electron Metamagnetism

**DOI:** 10.1038/srep08620

**Published:** 2015-03-02

**Authors:** D. P. Kozlenko, E. Burzo, P. Vlaic, S. E. Kichanov, A. V. Rutkauskas, B. N. Savenko

**Affiliations:** 1 Joint Institute for Nuclear Research, 141980 Dubna Moscow Reg., Russia; 2 Faculty of Physics, Babes-Bolyai University Cluj-Napoca 400084 Romania; 3 University of Medicine and Pharmacy “Luliu Hatieganu”, Physics Departament Cluj-Napoca, Romania

## Abstract

The itinerant electron metamagnetism (IEM) is an essential physical concept, describing magnetic properties of rare earth – transition metal (R-TM) intermetallics, demonstrating technologically important giant magnetoresistance and magnetocaloric effects. It considers an appearance of TM magnetization induced by spontaneous magnetization of surrounding R atoms, which provides significant response of the magnetic and transport properties on variation of external parameters (temperature, pressure, magnetic field) due to strong coupling between magnetic sublattices. The RCo_2_ compounds were generally considered as model systems for understanding of basic properties of IEM intermetallics. However, microscopic nature of magnetic properties still remains unclear. In our experimental and theoretical study of ErCo_2_ in a wide range of thermodynamic parameters a sequential collapse of cobalt sublattice magnetization in the background of nearly unchanged Er sublattice magnetization was revealed. The uncoupled magnetizations behavior challenges the IEM concept applicability and evidences more complex nature of magnetism in ErCo_2_ and related RCo_2_ systems.

The rare earth – transition metal intermetallic compounds R-TM (R = La, Pr, Nd…Lu, Y; TM = Co, Fe) exhibit a rich number of challenging physical phenomena, which were extensively studied during last years. They include itinerant electron metamagnetism, giant magnetoresistance and magnetocaloric effects as well as magnetoelastic lattice collapse^1^,^2^,^3^,^4^,^5^,^6^,^7^,^8^. The relatively simple RCo_2_ compounds exhibiting most of the aforementioned phenomena are considered as the best model systems for development and testing of theoretical concepts for the description of the properties of R-TM intermetallics and underlying driving mechanisms. At ambient conditions, they crystalize in the cubic structure of 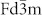 symmetry (so-called Laves phase), which undergoes structural distortions below the Curie points, T_c_, due to magnetostrictive effects^1^.

The magnetic ordering of Co sublattice in RCo_2_ compounds demonstrates extreme sensitivity to acting magnetic fields of internal (formed by surrounding magnetic ions) or external nature. The LuCo_2_^8^ and YCo_2_^1^ systems with non-magnetic R- element are exchange-enhanced paramagnets and cobalt magnetization can be induced only by application of strong external magnetic fields in the range H^c^ ≅ 70 to 75 T^9^. In RCo_2_ compounds with magnetic heavy rare-earths, the ferrimagnetic ordering with antiparallel orientation of the R and Co sublattices magnetization occurs, the cobalt magnetic moment is being of ≅ 1 μ_B_/atom at 4.2 K. The R-Co exchange interactions, mediating magnetic properties, are described in 4f-5d-3d model, through R5d band polarization, M_5d_^10^.

Originally Bloch et al^5^ analysed the magnetic properties of RCo_2_ compounds in the framework of the itinerant electron metamagnetism (IEM) concept^2^, considering conditions for a paramagnetic substance to become ferromagnetic by application and subsequent removal of a sufficiently strong magnetic field. Subsequently this concept, assuming correlated behavior of R and Co magnetizations was generally used for interpretation of the physical properties of RCo_2_ compounds. The value of internal magnetic field can be tuned by variation of R element or its content, substitution at cobalt sites^11^, application of external high pressure or magnetic field. The formation of the Co magnetic ordering leads to pronounced drop in resistivity of RCo_2_ compounds, and their macroscopic magnetic and transport properties were extensively studied during last years upon variation of R element type, external pressure and magnetic field^1^,^12^,^13^,^14^,^15^. However, microscopic mechanisms driving formation of magnetic properties remain unclear.

The good candidate to explore the microscopic nature of Co magnetization formation in RCo_2_ compounds and check the limits of the applicability of IEM concept is ErCo_2_, where the exchange interactions are moderate, as evidenced by the Curie temperature, T_c_ ≈ 35 K^1^,^8^,^12^. Several pressure studies were previously performed on ErCo_2_ and Er_1-x_Y_x_Co_2_ compounds using different experimental techniques. A decrease of Curie temperature with a rate *dT_c_/dP* = −8 K/GPa has been established in resistivity measurements on ErCo_2_ up to *P* = 2 GPa and it was attributed to pressure induced destabilization of the itinerant d system^16^. In subsequent resistivity studies over extended pressure range, it was found that *T_c_* stops to dimish and remains nearly constant at pressures above 4 GPa^12^. The X-ray adsorption spectroscopy (XAS) and X-ray magnetic circular dichroism (XMCD) studies evidenced a progressive reduction of Co magnetic moment as the applied pressure increases, but it is not canceled for *P* ≤ 4.2 GPa^17^. A remarkable reduction of the 3d spin moments upon compression was observed in ErCo_2_ in the magnetic Compton scattering experiments at pressures up to 1.84 GPa^18^. An asymmetry in *T_c_* – related ac susceptibility peak has been also observed under pressure and it was attributed to decoupling of the magnetic ordering of the Er and Co sublattices^19^.

In the studies of pseudobinary Er_1-x_Y_x_Co_2_ compounds Hauser et al. evidenced that the metamagnetic behavior of itinerant d subsystem vanishes for *x* > 0.4^20^. Two separate magnetic ordering temperatures were detected by means of specific heat, thermal expansion and electrical resistivity measurements. In contrast, using XCMD technique Chaboy et al^21^ reported that both Er and Co magnetic sublattices order at the same temperature for *x* ≤ 0.6. In particular, no decoupling of the magnetic ordering for both Er and Co sublattices has been shown for the compound with *x* = 0.4. The neutron diffraction study on Er_0.57_Y_0.43_Co_2_ compound, at *P* ≤ 0.6 GPa, evidenced the same Curie temperatures for both Er and Co sublattices, which decrease with a rate *dT_c_/dP* = −4 K/GPa^22^.

The above studies reported rather contradictory results. Also the collapse of the cobalt moment, as already mentioned, was suggested by indirect techniques as XAS, XMCD, magnetic Compton scattering, magnetic susceptibility or resistivity studies. In order to obtain more detailed and reliable information on magnetic properties of ErCo_2_ at microscopic level, we have performed neutron diffraction study of crystal and magnetic structure of ErCo_2_ compound in a wide range of thermodynamic parameters, high pressure 0–4 GPa and temperature 10–290 K, supported by extensive ab-initio theoretical calculations. While application of external high pressure allows to tune internal magnetic field value by reduction of interatomic distances, neutron diffraction measurements provide a possibility of simultaneous determination of both crystal and magnetic structure features in detail. We found that magnetizations of R and Co sublattices become uncoupled under pressure and the ordered Co magnetic moments collapsing in the background of nearly unchanged ordered Er magnetic moments.

## Results

### Experimental results

**Figure 1:**
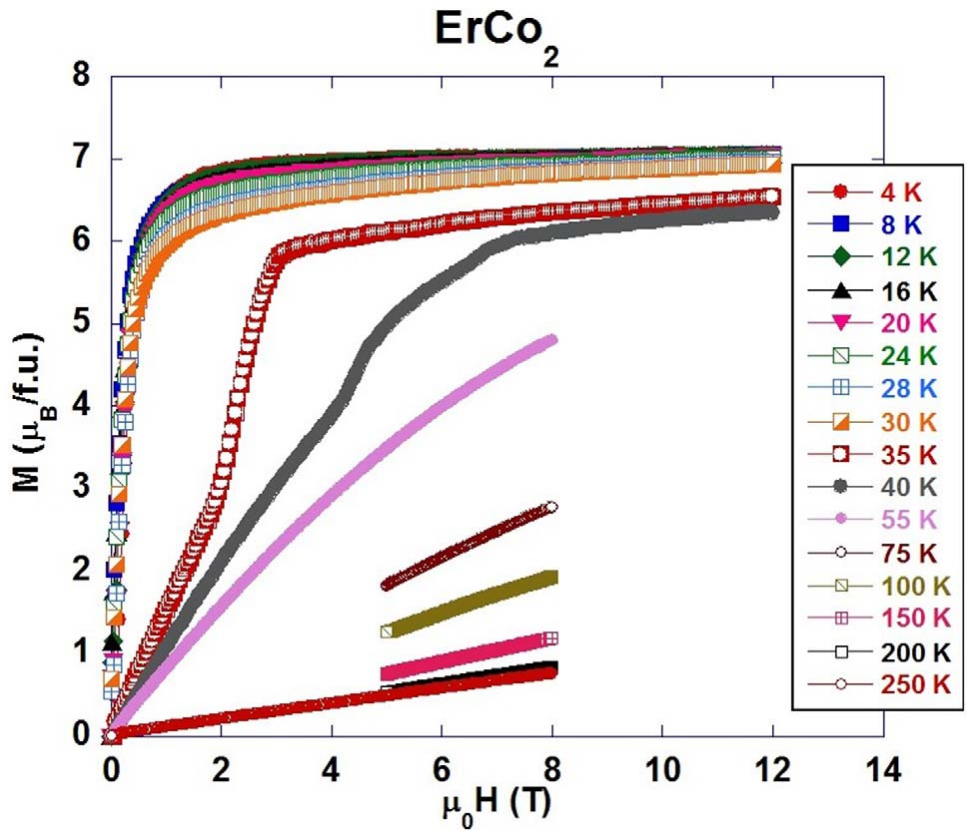
Magnetization isotherms for ErCo_2_ measured in external magnetic fields up to 12 T for selected temperatures in 4–250 K range.

**Figure 2:**
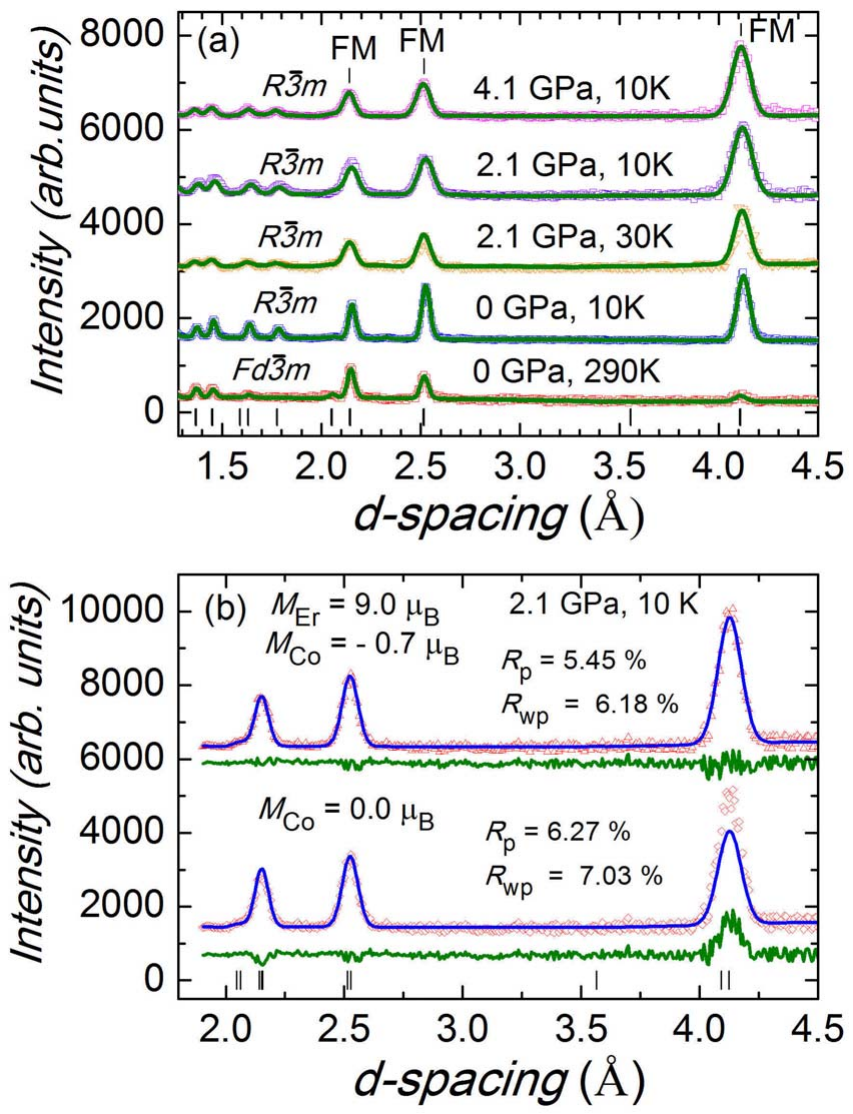
(a) Neutron diffraction patterns of ErCo_2_ compound, measured at selected pressures and temperatures and processed by the Rietveld method. Experimental points and calculated profiles are shown. Ticks below represent calculated positions of the structural peaks. The characteristic peaks with the most intense magnetic contribution are marked as “FM”. (b) The parts of neutron diffraction patterns measured at *P* = 2.1 GPa and *T* = 10 K, and calculated profiles for fixed Er magnetic moment M_Er_ = 9.0 μ_B_ and two values of Co magnetic moments M_Co_ = −0.7 μ_B_ and 0.0 μ_B_. The noticeable changes in difference curves demonstrate possibility of accurate determination of Co moments using neutron diffraction technique.

**Figure 3:**
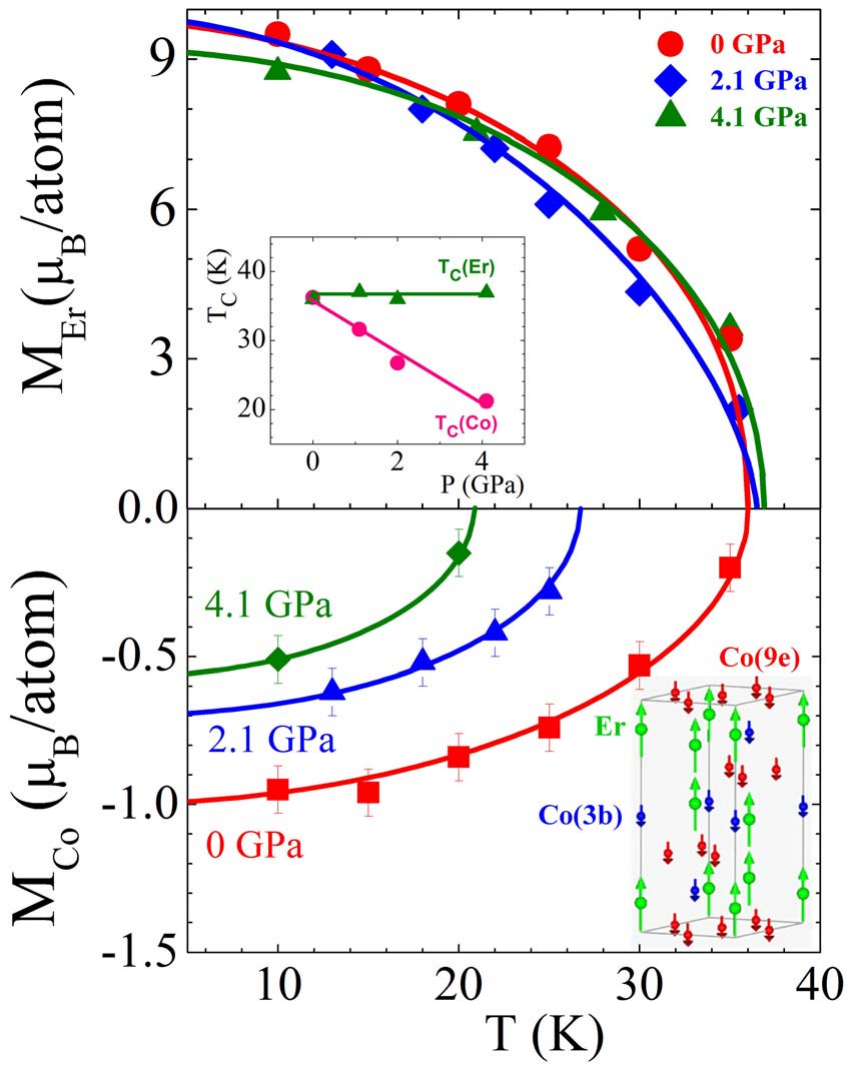
Temperature dependences of erbium and cobalt magnetic moments at pressures *P* = 0, 2.1 and 4.1 GPa and their interpolation by Brillouin functions. In the upper panel inset the calculated Curie temperatures *T_c_(Co)* and *T_c_(Er)* as a functions of pressure are presented. In the lower panel inset the crystal structure and ferrimagnetic arrangement of Er and Co moments is illustrated.

The magnetization isotherms, measured for ErCo_2_ compound, are shown in fig. 1. They demonstrate a presence of the first order magnetic transition at *T*_C_ ≈ 35 K, as previously reported^1^,^8^,^12^,^16^. The neutron diffraction patterns of ErCo_2_, measured below the Curie temperature (fig. 2), evidenced the rhombohedrally distorted crystal structure of 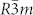 symmetry. There are two inequivalent positions for Co atoms, 9e and 3b, and one position, 6c for Er atoms in this structure, illustrated in fig. 3 (inset). The obtained structural parameters and ordered magnetic moments of Er and Co atoms at selected pressures and temperatures are presented in Table 1. The *a* and *c* lattice parameters decrease nearly linearly under pressure with compressibility coefficients *k*_a_ = 0.0051(9) and *k*_c_ = 0.0076(2) GPa^−1^ at 10 K. In the analysis of the magnetic contributions to neutron diffraction patterns at high pressures (fig. 2), we considered equal values of Co ordered magnetic moments located at positions 9e and 3b. It is supported by small difference in their values found from present data and previous studies^13^. While magnetization of Er sublattice provides major contribution to intensities of (1 0 1)/(−1 1 1), (1 1 0)/(2 −1 0) and (1 1 3)/(0 2 1)/(2 −1 3) peaks located at *d*-spacings *d*_hkl_ = 4.13, 2.53 and 2.16 Å, respectively, the magnetization of Co sublattice contributes mostly to the (1 0 1)/(−1 1 1) peak only. As a result, the changes in magnetization of Co sublattice can be determined precisely with respect to magnetization of Er sublattice, as illustrated by model calculations in fig. 2b.

The experimentally determined temperature dependences of the erbium and cobalt magnetic moments at different pressures are given in fig. 3 and their ferrimagnetic arrangement is illustrated in fig.3 (inset). The obtained magnetization per formula unit, *M*_fu_ ≈ 7.7 μ_B_, is close to one observed in magnetization data at low temperatures (fig. 1). The Curie temperature of erbium sublattice magnetization is little influenced by pressure, *dT_c_(Er)/dP* ≅0.3 K GPa^−1^ and very close to that of the whole ErCo_2_, compound at ambient pressure, *T_c_* ≈ 36 K (fig. 3, inset). The Er ordered magnetic moments at *T* = 10 K also remain nearly unchanged under pressure. In contrast, a collapse of the cobalt sublattice magnetizations at temperatures *T_c_(Co)*, which decrease with a rate *dT_c_(Co)/dP* = −(3.45 ± 0.3) KGPa^−1^, was evidenced (fig. 3). The ordered cobalt magnetic moments determined at 10 K as well as the extrapolated values at *T* = 0, decrease with a pressure rate *dM_Co_/dP* = −0.1 μ_B_GPa^−1^, similar to that in relevant RCo_2_ compounds (R = Tb, Ho)^14^,^15^. The relation 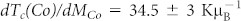, resulting from the data above, describes well the experimentally determined *T_c_(Co)* values – Table 2. These findings provide clear evidence of the magnetization collapse of Co sublattice under pressure at temperatures *T_c_(Co)* < *T_c_(Er)* and therefore, uncoupled behavior of Co and Er magnetic sublattices under pressure. The obtained results also imply that previously reported pressure dependences of the Curie temperatures^12^,^16^,^17^,^23^ for ErCo_2_ should be related to the collapse of cobalt sublattice magnetization only, characterized by *T_c_(Co)* and not to the total magnetization of the whole compound as reported.

The gradual collapse of cobalt magnetization, at temperatures *T_c_(Co)* can be analysed in correlation with temperature dependence of the exchange field, *H_ex_*, acting on cobalt atoms (*H_ex_* = 2*J*_CoCo_*M*_Co_ + *J*_CoEr_*M*_Er_). Starting from a two sublattices molecular field model^24^, the *J_ij_* (i,j = Er, Co) parameters describing the exchange interactions inside and between magnetic sublattices were evaluated using a fitting program^25^ of the magnetizations as determined by neutron diffraction. The values *J*_CoCo_ ≅ 95 and *J*_CoEr_ ≅ −5 were obtained. In case of heavy rare-earth RCo_2_ compounds, the *J*_R-Co_ values are linearly dependent on De Gennes factor, similar as their R5d band polarizations, as expected for a 4f-5d-3d exchange interaction model^10^. The thermal variations of exchange fields were computed assuming a Brillouin type dependence of cobalt magnetization and using the experimentally determined *M_Er_* values as illustrated in Fig. 3. At temperatures *T_c_(Co)*, where the cobalt magnetization collapses, the exchange field was 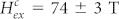. This value corresponds to the critical field necessary for supporting the cobalt ordered moment and it is nearly the same as that involved in inducing a cobalt ordered moment in LuCo_2_ by external field^9^, or by internal field in pseudobinary RCo_2_ compounds^26^,^27^,^28^. Conversely, assuming that the collapse of cobalt moment takes place at 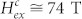, then *T*_c_(Co) values close to those experimentally determined were obtained. – Table 2.

### Theoretical calculations

**Figure 4:**
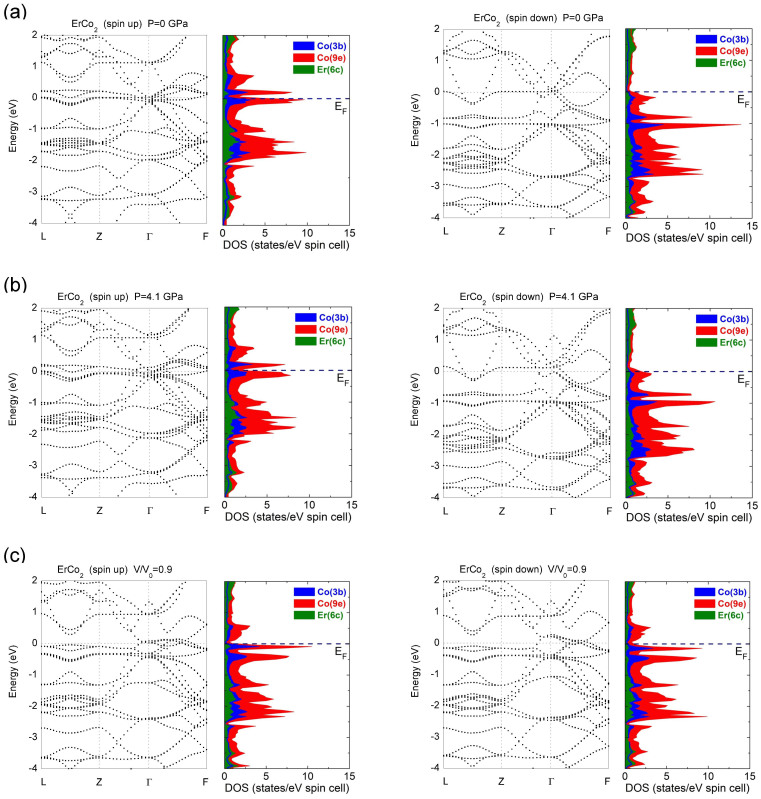
Total and partial densities of states of ErCo_2_ at pressures *P* = 0 GPa (v/v_0_ = 1) (a), 4.1 GPa (v/v_o_ = 0.97) (b) and v/v_o_ = 0.90 (c) (right part). In the left part the energy bands of ErCo_2_ decorated with orthogonal orbital character are shown. The location of the centers of the corresponding “fat” bands are only indicated. The contribution of Co3d and Er5d (d_xy_, d_yz_, d_zx_, 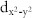, 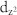), p (p_x_, p_y_, p_z_) and s orbitals to the “fat” bands were analysed as well as their evolution with pressure, relative volume, respectively on the energy scale. For some directions of the reciprocal lattice peculiar orbitals have dominant contributions, as mentioned in text.

**Figure 5:**
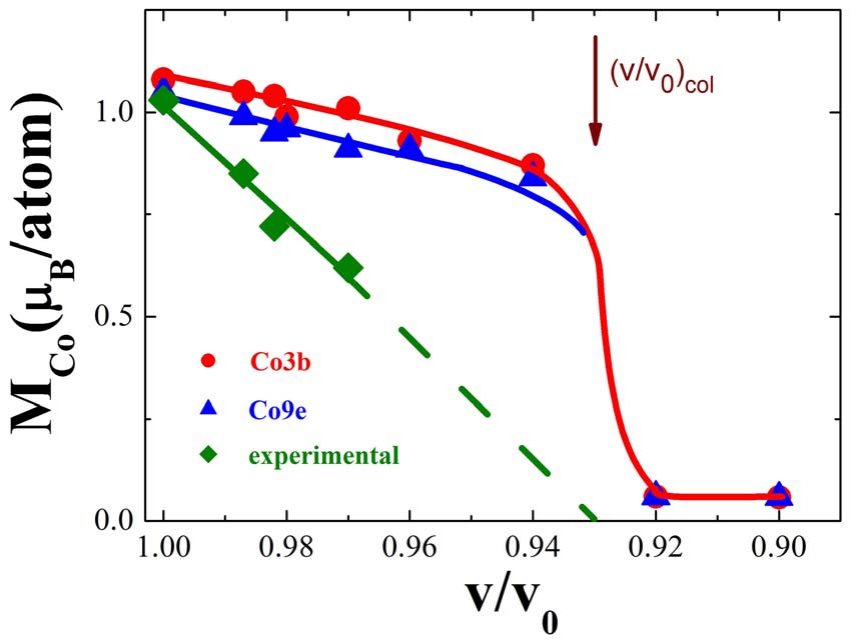
The dependence of cobalt moments at T = 0 K on relative volume change. The computed values for Co(9e) and Co(3b) moments and the experimental data extrapolated to T = 0 are given. The errors resulting from the refinements of experimental neutron diffraction patterns are of ±0.10 μ_B_.

The pressure evolution of cobalt moments at *T* = 0 K, was theoretically analysed in correlation with modification of the band structures – fig. 4. The computed cobalt moments at 3b sites are by ≅ 0.05 μ_B_ higher than those at 9e positions, but having the same trend as function of relative volume – fig. 5. The cobalt moments collapse rapidly at (*v/v_0_)*_col_ = 0.93. The experimentally determined *M*_Co_ values in the range *P* ≤ 4.1 GPa (*v/v*_0_ ≤ 0.97), decrease with a somewhat higher rate than those computed and exhibit more gradual collapsing trend. However, their extrapolation to *M*_Co_ = 0 gives about the same relative volume (*v/v_0_)*_col_ ≈ 0.93 as above, corresponding to the pressure value *P*_col_ = 9.5 GPa.

The band, formed by 5d electrons of erbium, Er5d, is found to be negatively polarized in ErCo_2_, as previously evidenced^10^. It contains main contribution from induced polarization by 4f-5d local exchange (determined when M_Co_ = 0), M_5d_(f) = −0.07 μ_B_, and also an additional contribution, M_5d_(0), due to 5d-3d hybridization. The M_5d_(0) values are proportional to the number of magnetic atoms, *z_i_*, situated in a first coordination shell to an Er one and their moments *M_i_*. The determined ratio *M_5d_(0)/*Σ*z_i_M_i_* = 2·10^−2^ is identical to that previously reported in RM_2_ (M = Fe, Co, Ni) heavy rare-earth compounds at ambient pressure^10^.

The analyses of band structures of ErCo_2_ in terms of projections of the bands onto orthogonal orbitals evidence some dominant contributions in peculiar directions of reciprocal lattice, mainly involved in pressure evolution of cobalt moments. In spin-up states, at *P* = 0 GPa, there are nondispersive features along Γ-Z direction near Fermi level with dominant contribution, from *d*_xz_ orbitals centered at ≅ 0.1 eV above *E*_F_, 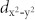 close to *E*_F_ and *d*_yz_ at 0.1 eV below *E*_F_. When increasing pressure, the above features shift to lower energies; that having mainly *d*_xz_ character, crossing the Fermi level at (*v/v_0_)*_col_ = 0.93. In spin down subband the nondispersive feature along Γ-Z direction located at −0.8 eV with dominant *d*_xz_ character, as well as that situated at −1 eV having mainly *d*_yz_ and 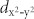 contributions (*P* = 0 GPa) shift to higher energies as effect of pressure, volume diminution, respectively. Thus, at the magnetic-nonmagnetic transition of cobalt the band initially located at −0.8 eV is close to *E*_F_.

When the nondispersive features having mainly *d*_xz_ character in the spin-up sub band cross *E*_F_, the density of states at *E*_F_ is large, as well as that corresponding to spin-down subband. As a result the Stoner's generalized stability condition^29^ is no more fulfilled and the cobalt magnetization at *T* = 0 K collapses.

## Discussion

The origin of Co magnetization collapse in ErCo_2_ is different from relevant intermetallic YCo_5_, where Co electronic configuration changes from high-to low spin state and accompanied by an isomorphic lattice collapse under pressure^6^,^7^. No anomalies in lattice parameters were detected in ErCo_2_ at temperatures T_c_(Co). This suggests that magnetovolume coupling is controlled mainly by erbium sublattice. The collapse of cobalt moments at *T* = 0 K, as effect of pressure, is well correlated with evolution of exchange splitting, *ΔE*_ex_ of their 3d band. There is a linear dependence of *M*_Co_ on *ΔE*_ex_, in agreement with previous data^30^,^31^. No changes in populations of the two Co3d subbands can be shown as effect of pressure.

In addition to itinerant metamagnetism model^2^,^5^, a model of induced magnetism has been also proposed, correlated with exchange field H_ex_ of external or internal nature, acting on cobalt atoms^27^,^32^. In the framework of this model, the cobalt magnetic moment is weakly dependent on the exchange field below critical value 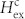 and it jumps to significantly higher value on approaching 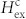. Upon further increase of the exhange field in the 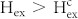 region a linear variation of cobalt moment followed by its saturation is expected. The cobalt moment has been correlated with the variation of the exchange splitting of their 3d band. Analysing the band structures of ErCo_2_, at different pressures, relative volume, respectively, there is a shift of spin up and spin-down sub-bands, respectively in opposite directions. The exchange splitting is thus diminished simultaneously with the decrease of cobalt moment. As showed previously^30^,^31^, there is a linear dependence of cobalt moment with the exchange splitting. No change in the population of the Co sub-bands is evidenced by band structure calculations, as required in the collective electron metamagnetism model. In addition, the cobalt moment is stable only in the presence of high external or exchange field. In all studies, particularly by neutron diffraction, a degree of localization of cobalt moment can be shown. Even in case of exchange enhanced LuCo_2_ paramagnet, at 100 K, in field of 5.72 T the density on Co atoms has a form factor which is similar to that of 3d electrons in Co metal^33^. Localized features for cobalt magnetism were reported also by NMR or other experiments^1^. These data show a more complex magnetic behavior than suggested by the model of collective electron metamagnetism. A better correlation with their 3d band exchange splitting, as function of the pressure is evidenced for ErCo_2_, as expected in the induced magnetism model.

The example of ErCo_2_ demonstrates the limits for the applicability of IEM concept for the case of RCo_2_ and relevant R-TM intermetallics with relatively low Curie temperatures and strength of R-Co interactions. In this case, decoupling of R and TM magnetization can be observed upon variation of thermodynamic parameters. This phenomenon is mediated by values of inter-sublattice exchange interaction *J*_R-TM_ and *R* 5d band polarization M_5d_(f), induced by 4f-5d local exchange and governing by DeGennes factor^10^. A reduction of the cobalt magnetic moment correlating with the behavior of the 3d band exchange splitting under pressure in ErCo_2_, is consistent with the induced magnetism model.

## Methods

### Experiment

The ErCo_2_ compound has been prepared by melting the high purity elements in an induction furnace, under high purity argon atmosphere. The sample was annealed at T = 880°C, in vacuum for one week. The electron microscopy and XRD studies, at ambient temperature, evidenced the presence of only one phase.

Magnetic measurements were performed in field up to 12 T, in a large temperature range. The errors in determining the magnetizations are around 0.06 μ_B_.

Neutron powder diffraction measurements, at P ≤ 4.1 GPa and T ≥ 10 K, were made with DN-12 spectrometer^34^ at IBR-2 high-flux pulsed reactor (FNLP Dubna, Russia) using the sapphire anvil pressure cell^35^. The pressure was determined by the ruby fluorescence technique with accuracy of 0.05 GPa, at each ruby chip and the pressure on the sample was obtained by averaging the values determined at different points. The data were analysed by Rietveld method using MRIA^36^ and Fullproof^37^ programs.

### Theory

The ground state electronic structures and magnetic properties of ErCo_2_ compound at various pressures, volume variations, respectively have been performed using scalar relativistic tight-binding linear muffin-tin orbital method (TB-LMTO) in atomic sphere approximation^38^. The local density approximation (LSDA) has been used for the exchange and correlation energy within von Barth and Hedin parameterization^39^. The valence basis consists of s-, p- and d- orbitals, whereas the Er4f orbitals were considered as core states. The overlaps of the muffin-tin spheres was below 9% and the standard combined corrections (CC) terms were introduced to compensate the errors due to ASA^38^. The band structures were computed starting from lattice parameters determined at 10 K and pressures of 0, 1.1, 2.1 and 4.1 GPa. The corresponding normalized volumes were v/v_0_ = 1.0, 0.989, 0.982 and 0.97, respectively. A sample with v/v_0_ = 0.90 has been also analysed. The minimum total energy has been obtained for a and c lattice parameters which differ from experimentally determined values by ≅ 0.6%.

## Author Contributions

D.P.K. and E.B. prepared the main manuscript text and display items. P.V. performed ab-initio calculations. E.B. performed magnetization measurements. S.E.K. and A.V.R. performed ND experiments. A.V.R. and B.N.S. made a preliminary ND data treatment. All authors reviewed the manuscript.

## Figures and Tables

**Table 1:** Structural parameters and ordered magnetic moments of Er and Co atoms in ErCo_2_ at selected pressures and temperatures. In the ambient temperature cubic 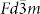 structure Er atoms locate at sites 8(a) (0.125, 0.125, 0.125) and Co atoms locate at sites 16(d) (0.5, 0.5, 0.5). In the low temperature rhombohedral 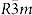 structure Er atoms locate at sites 6(c) (0, 0, *z*) and Co atoms locate at sites 3(b) (0, 0, 0.5) and 9(e) (0.5, 0, 0). The reliability *R_p_* and *R_wp_* factors values are also given

*P* (GPa)	0		2.1		4.1	
*T* (K)	290	10	290	10	290	10
Space group	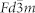	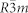	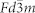	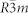	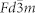	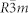
*a* (Å)	7.1115(6)	5.0495(4)	7.0713(7)	5.0419(7)	7.0492(7)	5.0337(7)
*c* (Å)	-	12.307(7)	-	12.126(8)	-	12.027(8)
Er *z*	-	0.1238(5)	-	0.124(1)	-	0.122(1)
*B*_iso_ (Å^2^)	0.32(6)	0.20(6)	0.30(6)	0.17(8)	0.29(6)	0.16(8)
*M_z_* (μ_B_)	-	9.60(8)	-	9.0(1)	-	8.8(1)
Co *B*_iso_ (Å^2^)	0.43(7)	0.30(7)	0.41(7)	0.28(8)	0.38(6)	0.26(8)
*M_z_* (μ_B_)	-	−0.95(5)	-	−0.70(7)	-	−0.50(7)
*R*_p_, %	4.72	4.93	5.38	5.45	5.73	5.93
*R*_wp_, %	5.21	5.42	6.03	6.18	6.28	6.42

**Table 2:** The Curie temperatures of cobalt sublattice magnetization

	T_c_(Co) (K)			
Method	P = 0 GPa	P = 1.1 GPa	P = 2.1 GPa	P = 4.1 GPa
Experimental	35 ± 0.5	29.5 ± 1.0	27 ± 0.5	21.5 ± 0.5
T_c_(Co) = 34.5 M_Co_	34.9 ± 0.4	28.6 ± 0.3	24.8 ± 0.4	21.1 ± 0.3
From critical value of exchange field, 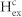 = 74 T	32 ± 2	30.5 ± 0.7	25 ± 0.8	22.0 ± 0.5
